# Nature-Inspired Hierarchical Steels

**DOI:** 10.1038/s41598-018-23358-7

**Published:** 2018-03-23

**Authors:** Shan Cecilia Cao, Jiabin Liu, Linli Zhu, Ling Li, Ming Dao, Jian Lu, Robert O. Ritchie

**Affiliations:** 1 Department of Materials Science & Engineering, University of California, Berkeley, California, 94720 USA; 2 Department of Mechanical & Biomedical Engineering, City University of Hong Kong, Hong Kong, China; 3 Department of Materials Science & Engineering, Massachusetts Institute of Technology, Massachusetts, 02139 USA; 4 Center for Advanced Structural Materials, City University of Hong Kong Shenzhen Research Institute, Shenzhen, 518000 China; 5 School of Materials Science and Engineering, Zhejiang University, Hangzhou, 310027 China; 6 Department of Engineering Mechanics, Key Laboratory of Soft Machines and Smart Devices of Zhejiang Province, Zhejiang University, Hangzhou, 310027 China; 7 Department of Mechanical Engineering, Virginia Tech, Blacksburg, VA 24061 USA; 8 Materials Sciences Division, Lawrence Berkeley National Laboratory, Berkeley, California, 94720 USA

## Abstract

Materials can be made strong, but as such they are often brittle and prone to fracture when under stress. Inspired by the exceptionally strong and ductile structure of byssal threads found in certain mussels, we have designed and manufactured a multi-hierarchical steel, based on an inexpensive austenitic stainless steel, which defeats this “conflict” by possessing both superior strength and ductility. These excellent mechanical properties are realized by structurally introducing sandwich structures at both the macro- and nano-scales, the latter via an isometric, alternating, dual-phase crystal phases comprising nano-band austenite and nano-lamellar martensite, without change in chemical composition. Our experiments (transmission and scanning electron microscopy, electron back-scattered diffraction, nano-indentation and tensile tests) and micromechanics simulation results reveal a synergy of mechanisms underlying such exceptional properties. This synergy is key to the development of vastly superior mechanical properties, and may provide a unique strategy for the future development of new super strong and tough (damage-tolerant), lightweight and inexpensive structural materials.

## Introduction

In acquiring high strength, metallic materials are invariably compromised by low ductility and toughness^1–4^. As structural materials for most engineering applications require all these often mutually-exclusive properties, the design of such metallic alloys frequently represents a compromise^5–7^. Nature, however, is particularly adept at developing damage-tolerant materials that are both strong and ductile through the creation of hierarchical (multi-scale) architectures with gradients in structure and properties^8,9^. Accordingly, natural and biological materials have provided much inspiration over the past decade in the quest for new and improved structural materials^8,10,11^; this has been especially true in the study of marine species which tend to be strong and tough in order to survive in their complex environment^8,12–14^. One such material is byssus, a series of byssal threads made of fibrous proteins, which are conceived to be strong^15,16^, with tensile strengths of ~120 MPa, yet are sufficiently ductile and extensible (uniform elongations ~80%) to act as climbing ropes for the mussel.

**Figure 1:**
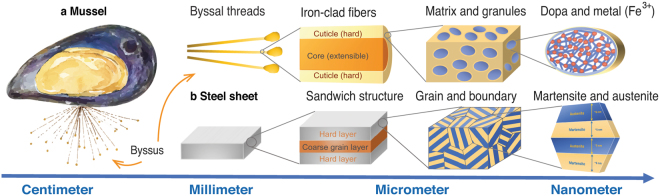
Design concept: the overall architecture of the hierarchical steel, as compared to that of strong and tough natural byssal threads. (**a**) Sandwich structures of byssal threads and (**b**) the hierarchical steel developed in this work. On the micro-scale, the threads are made of a matrix and granules which vary in cross-linking density, the granules containing a higher cross-link density than the matrix; this controls the difference in local mechanical properties. From the micro-scale point of view, the hierarchical steel is comprised of a hard layer and a coarse-grained layer. At the nano-scale, there are two compositions, dopa and metal (Fe^3+^), that comprise a single granule. At the same dimension, our hierarchical steel has a graded dual-phase structure consisting of nano-scale martensite and nano-scale austenite, which are distributed as alternating lamellar, ~5 to 6 nm thick, inside each grain of the steel.

Akin to most natural materials, the superior combination of mechanical properties of byssal threads result from their hierarchical and graded ultrastructure, as shown in Fig. 1a. The structure of each hierarchy is made of two kinds of protein complexes with different density of cross-linking. In particular, the concentrated cross-linking in the granules provides hardness, whereas the less cross-linked matrix provides extensibility^16^. The integration of such structural features is the key to the attainment of superior mechanical performance in byssal threads, which is also the goal of this bio-inspired design. We hypothesized that a similar mechanical response could be transferred to structural steels by designing a micro-scale sandwiched architecture to benefit from the creation of critical structural features over multiple length-scales^5,17,18^. Specifically, we took an inexpensive and lightweight austenitic stainless steel and introduced a nano-scale, dual-phase structure of martensite and austenite (similar to Fe^3+^ and dopa in byssus) at the sub-grain level by controlling the nano-structure through processing^19^, described in the Methods section of the Supplementary Information. Samples were first annealed and then underwent a carefully-controlled surface nano-crystallization (SNC) technology (also described in the Supplementary Information Methods section). These finely-controlled treatments resulted in a multi-hierarchical steel possessing the potent combination of two specific characteristics: a multi-scale sandwiched architecture with a nano-scale dual-phase lamellar structure. Figure 1b illustrates the hierarchical steel developed in this work. From the micro-scale point of view, this steel is comprised of a hard layer and a coarse-grained layer. At the nano-scale, it has a graded dual-phase structure consisting of nano-scale martensite and nano-scale austenite, which are distributed as alternating lamellar, ~5 to 6 nm thick, inside each grain of the steel.

**Figure 2:**
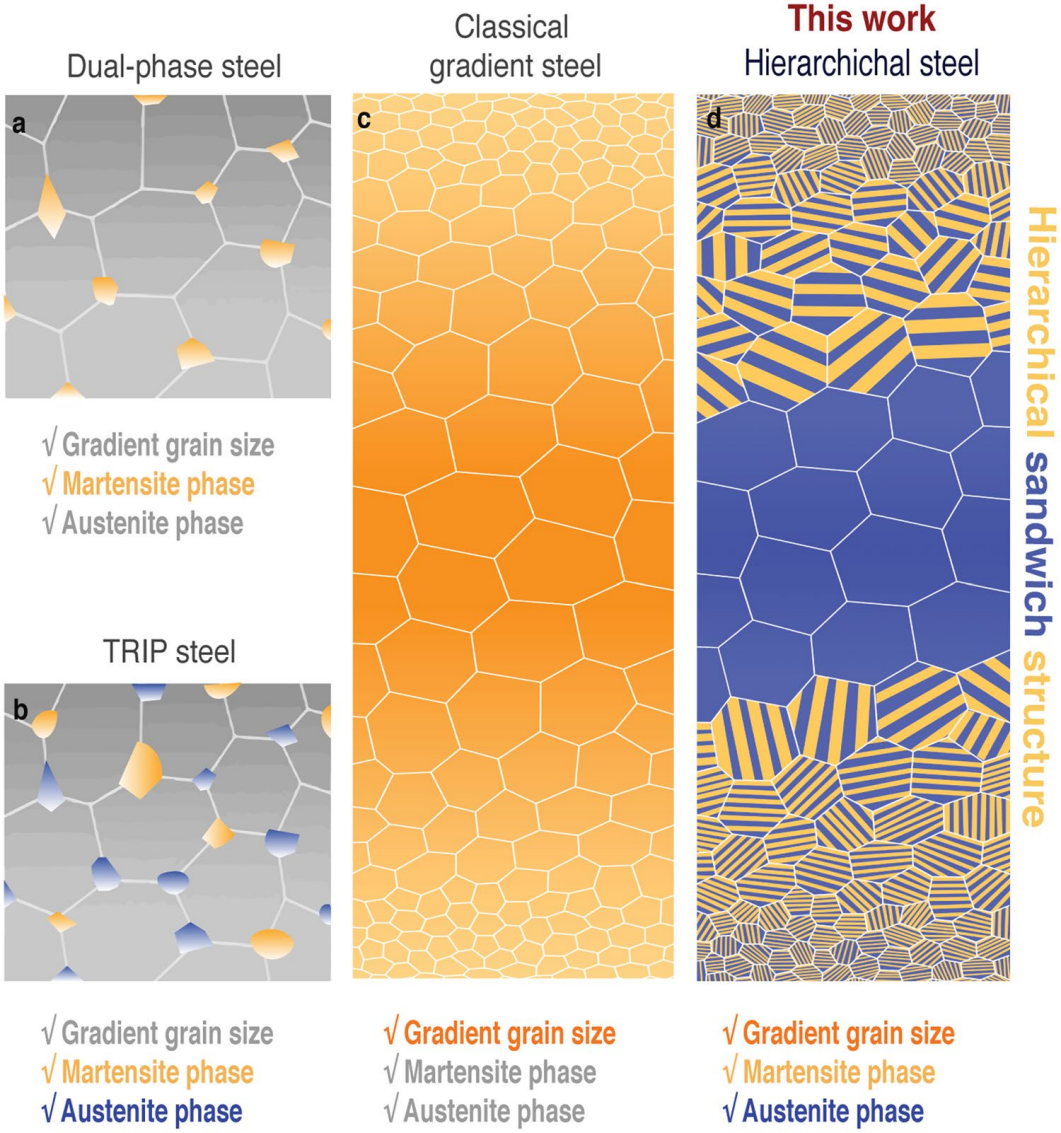
The overall structure of the hierarchical steel as compared to that of other strong and tough steels. (**a**) Schematic illustrations of the microstructure of a dual-phase steel showing the martensite phase (yellow lumps) randomly embedded in the ferrite matrix. (**b**) TRIP steel showing the martensite (yellow lumps) and austenite (blue plates) phases embedded in the ferrite matrix; note that there are no gradient structures in either the dual-phase or TRIP steels. (**c)** classical gradient steel displaying a traditional gradient in grain size. (**d)** our hierarchical steel showing a sandwich structure of nano-scale dual-phase (martensite and austenite) in the surface region (extending some 300 µm from the surface) with a coarser-grained austenitic region in the core of the steel.

**Figure 3:**
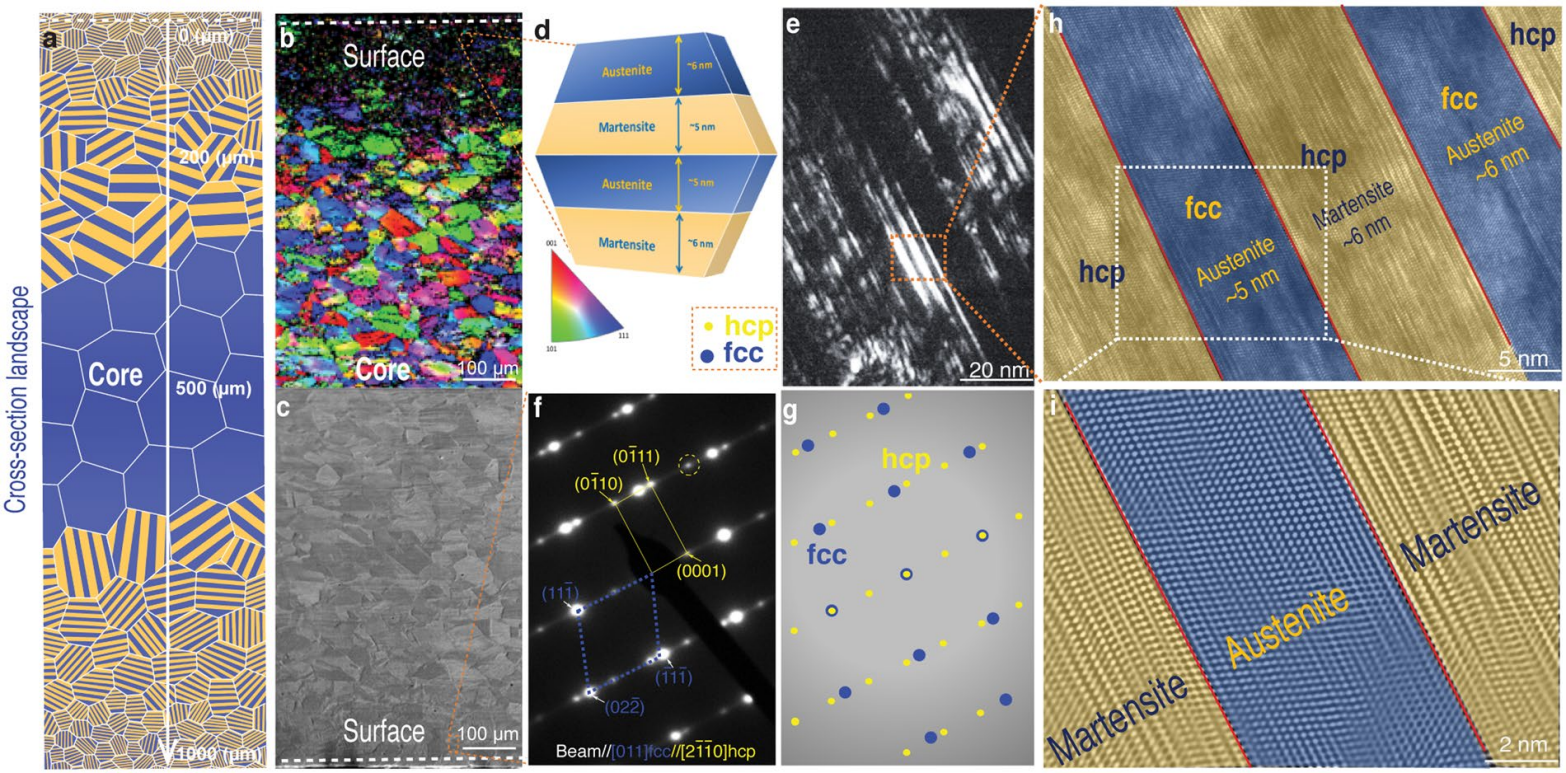
Multi-scale structural characterization of the hierarchical steel. (**a**) Schematic of the gradient in grain size distribution of our hierarchical steel with depth from one topmost surface (outlined by dashed line) to the lower surface of the steel (~1000 µm in total). (**b**) The EBSD phase map and (**c**) SEM micrograph across the cross-section. (**d**) Nano-sized martensite and austenite phase distribution in one typical grain. Grain sizes are about 15 to 20 nm at the topmost surface, outlined by dashed line in (**b)**,(**c**) and (**e**). A typical dark-field TEM image taken at ~100 nm deep underneath the surface of our steel where bright area/bands are ε-hcp martensite. (**f)** The SAED patterns corresponding to (**e)** clearly show the combination of diffraction spots from both austenite and ε-hcp martensite. (**g**) Schematic illustration of the SAED patterns of (**e)** The relatively weak spots (yellow spots) are from the ε-hcp martensite, with the axis of $[2\bar{1}\bar{1}0]$ hcp, and the relatively strong spots (blue spots) are from the austenite, with the axis of [011] fcc. The austenite has the following orientation relationship with the ε-hcp martensite: [011] fcc and $[2\bar{1}\bar{1}0]$ hcp, and $(\bar{1}\bar{1}1)\text{fcc}//(0002)\text{hcp}$. (**h)** shows a high-resolution image of the dash box selection in (**e)** which illustrates the alternative arrangement of the two nano-scale phases: martensite and austenite (both with thicknesses of ~5 to 7 nm). The nano-lamellar martensite produced by strain-induced phase transformation from austenite matrix. (**i**) shows an IFFT image corresponding to the selection in (**h**).

Can such a designed and tunable hierarchical steel be both strong and ductile? To address this question, we first investigate the microstructure. We first compare our bio-inspired hierarchical steel, which exhibits a unique combination of structural features, with current advanced high-performance steels (Fig. 2): (a) a dual-phase (DP) steel, comprising typically stable ferrite and martensite^20,21^, (b) transformation-induced plasticity (TRIP) steels, where the austenite phase is unstable and can undergo a stress-induced transformation to martensite^22^, and (c) classical gradient steels (a group of steels those are characterized by a systematic change in microstructure on a macroscopic scale), which typically possess a gradient in grain size and hardness, and (d) our hierarchical steel, with the added nanoscale lamellar structure. The hierarchical steel combines the particular attributes of the three other steels, including a gradient in grain size from micro- to nano-meter scales and a duplex structure of nano-lamellar martensite and nano-band austenite phases that are confined to within ~300 µm of the surface (Fig. 3a). It has been shown that the gradient in grain size - the finer grained-structure at the surface promotes a hard surface layer, without compromising the ductility of the bulk – can serve to negate the strength-ductility trade-off by enhancing the yield strength and promoting strain-hardening which in turn can enhance ductility by delaying the onset of the maximum-load instability (necking)^17,23–25^.

**Figure 4:**
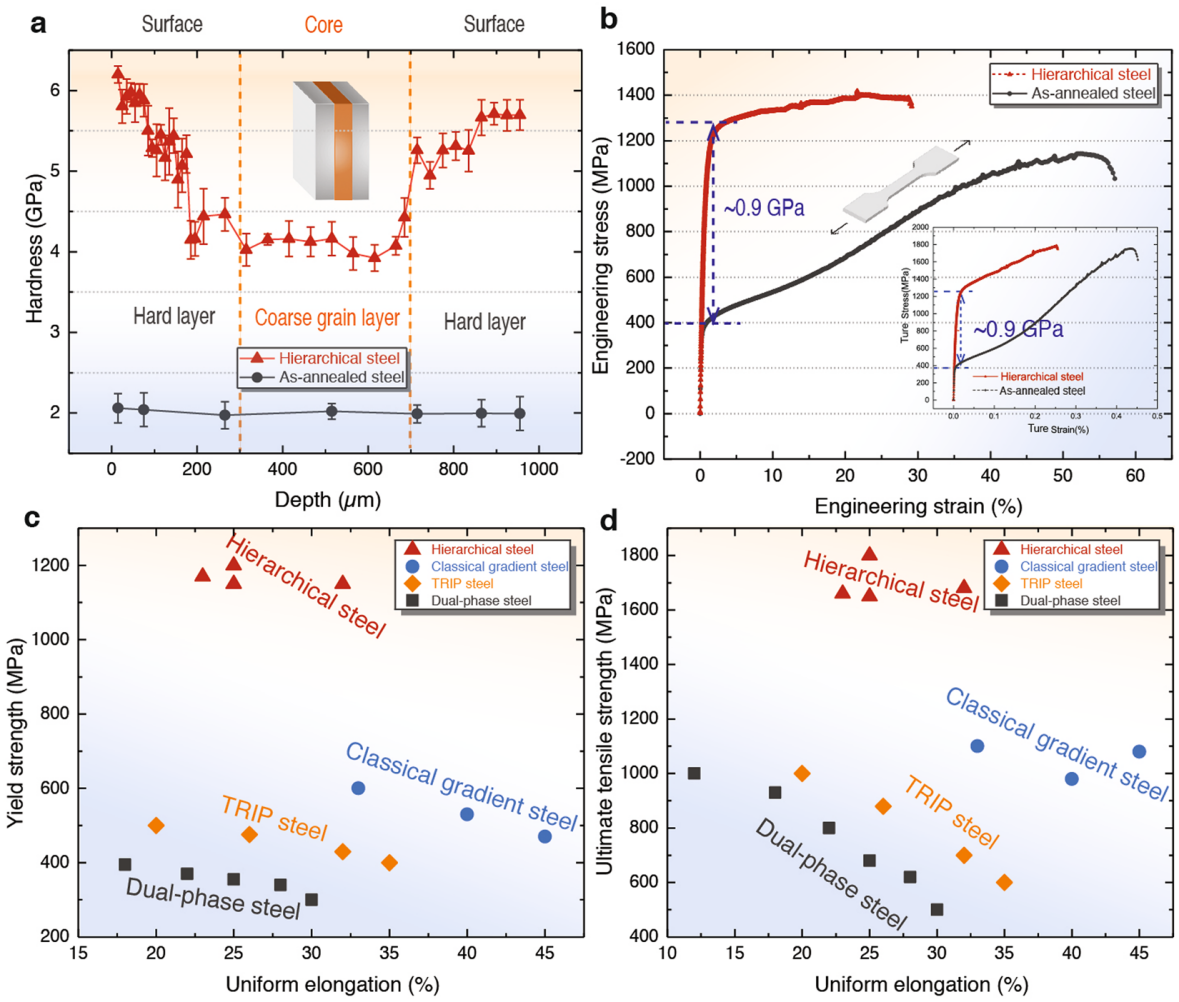
Comparison of mechanical properties of the hierarchical steel with those of other steels. (**a**) Hardness distribution along the cross-section of an as-annealed steel, as compared to our hierarchical steel. Results indicate that the hierarchical steel (red scattering) attains larger improvements in hardness as compared to as-annealed steels. (**b**) Uniaxial tensile tests show engineering stress–strain curves of the hierarchical steel (in red triangle scattering), as compared with that of as-annealed steel; inset graphs show the corresponding true stress-strain curves. Representative tensile properties of steels are shown in (**c**) and (**d**). The data are for dual-phase steels, TRIP-steels, classical gradient steels and our hierarchical steel (this work). Red triangle data points represent the tensile properties of the hierarchical steel. A comparison of the respective yield (**c**) and ultimate tensile strengths (**d**) of dual-phase steels, TRIP-steels, classical gradient steels and hierarchical steels, indicates that the hierarchical steels have a far superior combination of strength and ductility in comparison to other steels.

Inspired by strong and ductile structure in byssal threads, we sought to mimic its hierarchical structure. Our rationale here was to design structural features into the hierarchical architecture of this steel to make it exceptionally strong and ductile in comparison to other steels in order to diversify its application as a structural material. Its uniaxial tensile properties are shown in Fig. 4, where it can be seen that it displays a superior combination of strength and ductility compared to the DP steel, TRIP steel and classical gradient steel. Additionally, the cross-sectional hardness distribution in Fig. 4a indicates that our hierarchical steel is much harder than as-annealed steels, measured at the same depth below the surface.

**Figure 5:**
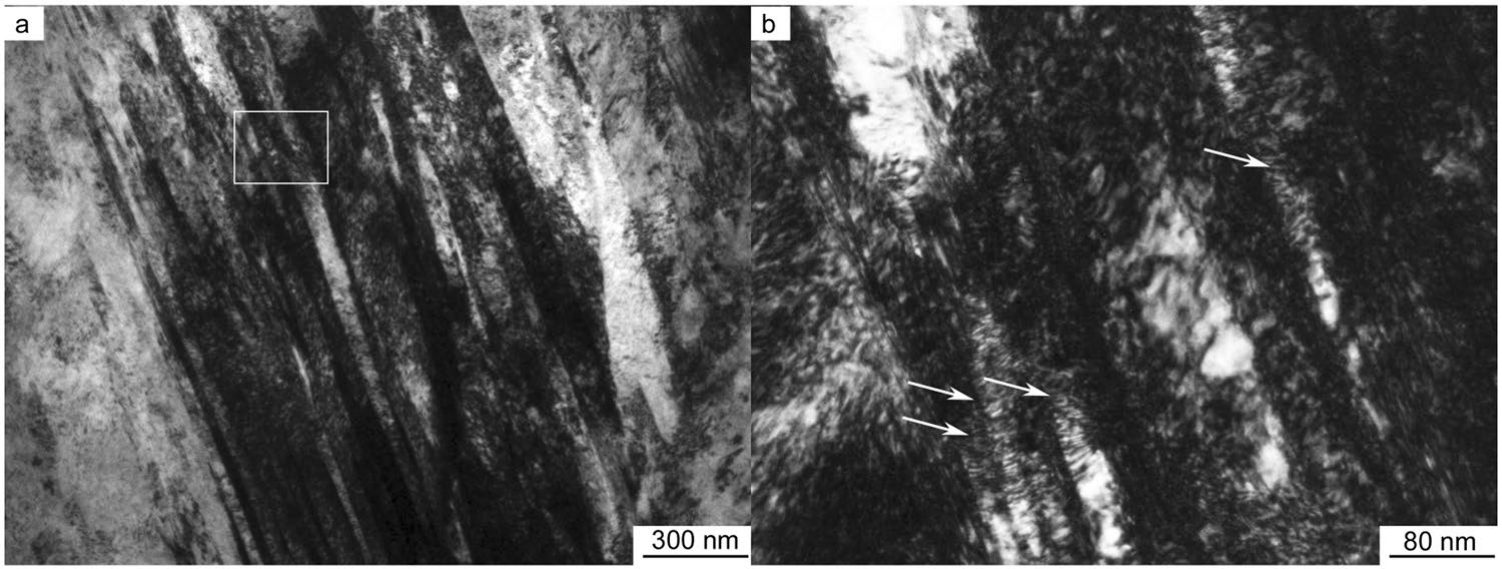
Microstructure after tensile test (**a**) TEM image of the tensile sample near the fracture zone showing high density of dislocations and (**b**) magnified zone of the white rectangle in (**a**), showing the numerous dislocation pile-ups.

The question that immediately arises is why this hierarchical steel, featuring a gradient in grain size on the micro-scale and an alternate arrangement of austenite and martensite on the nano-scale, exhibits such improved hardness, yield and tensile strength, and uniform tensile elongation, as compared to the other steels? We believe that this is associated with the unique synergy of deformation mechanisms created in this steel, as shown by the scanning electron microscopy (SEM), electron back-scatter diffraction (EBSD) and transmission electron microscopy (TEM) images in Fig. 3, together with the mechanism-based constitutive modeling described in the Supplementary Information Methods section. A cross-section landscape of the SEM image in Fig. 3c together with the EBSD phase map in Fig. 3b reveal the gradient in grain size from the surface to the core in the hierarchical steel, respectively at the nano- and micro-scales. Specifically, the grain size gradient can be seen to vary from ~15 to 20 nm at the topmost surface (near dashed line area in Fig. 3c) and increases gradually to the microscale with increasing depth. Furthermore, the inside of each grain is comprised of nano-scale alternating layers of martensite and austenite (Fig. 3d), with lamellar thicknesses ranging from 5 to 7 nm at the topmost surface, as outlined by the dashed lines in Fig. 3b,c. This alternating martensite/austenite nano-lamellar structure within the grain is shown in greater detail in the TEM images in Fig. 3e,h,i; specifically, the dark-field TEM image in Fig. 3e reveals this dual-phase nanostructure at ~100 nm beneath the surface of the steel. Using corresponding selected area electron diffraction (SAED), shown in Fig. 3f, the bright nano-bands were identified to be hexagonal closed-packed (hcp) ε-martensite, having the following orientation relationships with the face-centered cubic (fcc) γ-austenite: [011] fcc // [$2\bar{1}\bar{1}0$] hcp and ($\bar{1}\bar{1}1$) fcc // (0002) hcp, the ε- martensite forming by a stress-induced phase-transformation from the γ-austenite matrix. In Fig. 3f, the SAED patterns, corresponding to Fig. 3e, clearly show the combination of diffraction spots from both austenite and ε-hcp martensite. At the same time, Fig. 3g is the schematic illustration of the SAED patterns where the relatively weak spots (yellow spots) emanate from the ε-hcp martensite, with the axis of [$2\bar{1}\bar{1}0$] hcp, whereas the relatively strong spots (blue spots) are from the austenite, with the axis of [011] fcc. A high-resolution image of the morphology and distribution of these dual nano-scale phases is shown in Fig. 3h; both the austenite and martensite lamellar have thicknesses of ~5 to 7 nm, as confirmed by the Inverse Fast Fourier Transition (IFFT) image in Fig. 3i. The thickness of the ε-bands varies from 5 to 7 nm and are homogeneously dispersed among the austenite. High-resolution TEM images in Fig. 3h,i clearly show the atomic arrangement within the austenite and ε-hcp martensite. The interface between the two phases is parallel with (111) fcc and (0002) hcp, with the packing sequence of the most closed-packed plane being ABCABC in the austenite and ABAB in the ε-hcp martensite. Overall, the alternating nano-scale lamellar austenite and martensite architecture with its innumerable interfaces inside each grain form a local nano-/micro-scale structural unit which serves as an effective source, sink, as well as a strong barrier to dislocation motion during deformation. Such a dual-phase lamellar nanostructure inside the grains provides an essential distinction of this hierarchical steel with its superior mechanical properties to that of classical gradient steels. At the same time, the hierarchical steel possesses a coarser-grained austenitic interior which acts as a “softer extensible core”, in the same manner as in byssal threads, and contributes to the excellent ductility of the steel. Taken as a whole, the hierarchical nano-/microscale sandwich/dual-phase structure results in an unprecedented combination of strength and ductility, the often mutually exclusive prime mechanical properties of a structural material. In addition, we believe that the microstructure after tensile testing provides a useful means to fully understand the deformation mechanisms. This is shown in Fig. 5 where TEM images of the microstructure are presented from samples which were extracted near the fracture zone of the tensile samples. An extremely high density of dislocations can be seen in Fig. 5a, with numerous pile-ups of dislocations between the interfaces indicated by the arrows in Fig. 5b. This observation strongly supports our hypothesis that the nano-lamellae formed by the martensite are effective blocks to impede the movement of dislocations.

The essence of a structural material is its mechanical properties, which is the embodiment of its microstructure^26–30^. In terms of numerical values, our hierarchical steel displays respective yield and tensile strengths of 1.2 and 1.8 GPa at ambient temperatures, while retaining a tensile ductility of 25% during uniaxial tensile tests (Fig. 4b). These excellent mechanical properties are realized by structurally introducing sandwich structures at both the macro- and nano-scales, the latter via an isometric, alternating, dual-phase crystal phases comprising nano-band austenite and nano-lamellar martensite. Dual-phase grains at the surface serve as potent sources of dislocation motion during deformation which contributes to its high strength, while in the core, the austenite structure can act as a relatively extensible core, enhancing ductility for the entire structure. Cross-sectional nano-indentation hardness measurements translated across the ‘sandwich structure’ of the steel indicate hardness values varying from ~6.3 GPa at surface to ~4.2 GPa in the core with indentation depth of ~2.5–3.2 µm (Fig. 4a). These damage-tolerant properties exceed those of other steels, such as DP steel, TRIP-steels and even prevailing classical gradient steels, insomuch that our hierarchical steel has both superior strength and hardness with comparable ductility (Fig. 4c,d). As noted above, this follows because the grain boundaries and especially multiple austenite-martensite interfaces within the nano-scale dual-phase grains at the surface layers (to a depth of ~300 μm) serve as potent sources of the retardation of dislocation motion during deformation, resulting in high strength, whereas the coarser-grained austenite structure at the center of the steel can act as a relatively soft extensible core, promoting ductility throughout the entire structure.

To provide support to these ideas and our corresponding experimental results, a theoretical model was developed, the details of which are described in the Theoretical Modeling section of the Supplementary Information. The calibrated model (Supplementary Figure 1 and related text) is formulated based on the multiple length-scale structure of the hierarchical steel; specifically, the nano-lamellae formed by martensite is deemed to act as effective blocks to impede the movement of dislocations inside each grain, leading to dislocation pile-ups along the martensite/austenite interfaces. Accordingly, dislocation pile-up zones are created near the interfaces between the nano-lamellar martensite and nano-lamellar austenite in the model. The yield strength (and/or flow stress) of the material can then be directly related to the dislocation density of these zones (Supplementary Figure 2a, b), which depends on the depth-dependent thickness of nano-lamellae and the grain size of the grains (see insets in Supplementary Figure 1). In general, the thinner the lamellae, the higher the dislocation densities and the flow strength. Therefore, nano-lamellar structures near the surface layers possess much higher load-bearing capacity and consequently much higher local strength compared with the companion classical gradient steel without nano-lamellae (Supplementary Figure 2c). For example, due to the much higher average flow strength, the outermost layer (with a depth below the surface of ~100 µm), with the smallest nano-lamellae occupying only 20% of the overall volume, can contribute more than 50% of the overall load under uniaxial tension (Supplementary Figures 3a,b); the nano-lamellae layer (with a depth below the surface of ~300 µm) also has ~400 MPa higher average strength than the comparable classical gradient steel with the same grain-size gradient (Fig. 3c). In addition, the experimentally observed (and theoretically simulated) smooth, through-thickness variations in yield strength, hardness and microstructure size-scale (Supplementary Figure 1) can prevent significant stress/strain jumps within the gradient structure, thereby avoiding sharp interfaces which can induce stress and strain concentrations. The synergetic effect between macro-scale and nano-scale landscapes of our hierarchical steel serves as the principal feature to defeat the strength-ductility trade-off to result in the exceptional mechanical performance of the steel.

To conclude, we recognize that Nature took billions of years to craft the sandwich structure of marine byssal threads which represent a remarkable dual-phase material combining excellent strength with high ductility. This natural structure combines the two essential features of natural structural materials, namely a hierarchical architecture spanning multiple length-scales together with effective gradients in structure and properties, all designed to confer specifically functional performance. We have merely mimicked this a masterpiece of Nature’s evolution yet believe that the graded hierarchical system with a nanoscale dual-phase structure that we describe here, which we demonstrate yields unprecedented damage tolerance, can be applied to the design of other inexpensive materials in the search for superior structural materials for the future. We trust that this work may serve as a yet another step in the quest of developing new lightweight structural materials using the inspiration of natural and biological materials.

## Electronic supplementary material

Supplementary Information
