# Supplementary material for: Nature-Inspired Hierarchical Steels
Source: Sci Rep. 2018 Mar 23;8:5088. doi: 10.1038/s41598-018-23358-7 (PMC5865137; doi:10.1038/s41598-018-23358-7)
Supplement: Supplementary file 1 — Supplementary Information [file 41598_2018_23358_MOESM1_ESM.pdf]

Supplementary Information

Nature-Inspired Hierarchical Steels

Shan Cecilia Cao[1,2,3], Jiabin Liu[2,5], Linli Zhu[6], Ling Li[7], Ming Dao[3], Jian Lu[2,4], Robert O. Ritchie[1,8]

[1] Department of Materials Science & Engineering, University of California, Berkeley, California 94720, USA

[2] Department of Mechanical & Biomedical Engineering, City University of Hong Kong, Hong Kong, China

[3] Department of Materials Science & Engineering, Massachusetts Institute of Technology, Massachusetts 02139, USA

[4] Center for Advanced Structural Materials, City University of Hong Kong Shenzhen Research Institute, Shenzhen 518000, China

[5]School of Materials Science and Engineering, Zhejiang University, Hangzhou, China

[6] Department of Engineering Mechanics, Key Laboratory of soft machines and smart Devices of Zhejiang Province, Zhejiang University, Hangzhou 310027, China

[7] Department of Mechanical Engineering, Virginia Tech, Blacksburg, VA 24061, USA.

[8] Materials Sciences Division, Lawrence Berkeley National Laboratory, Berkeley, California, 94720, USA

Methods

The material used in this work was an austenitic AISI 301 stainless steel with a chemical composition (in wt.%) of 16.6 Cr, 6.2 Ni, 0.4 Mo, 0.13 C, 0.45 Si, 1.7 Mn, 0.03 S, 0.03P and the balance Fe. Samples were cut into plates, 110 × 50 ×1 mm^3 in size, for heat treatment and surface nanotechnology. Samples were annealed *in vacuo* at 1150°C for 2 h and then quenched into water, prior to both surfaces of the specimens being treated by a Surface Mechanical Attrition Treatment (SMAT) process[31,33-35]. The SMAT process is essentially a dynamic plastic deformation process. The steel plates were placed on the top side of a chamber which contains hundreds of hard balls with a diameter of 3 mm. Those balls were vibrated using high-power ultrasound so that they impacted onto the surface of the steel plates at a high speed. Essentially, the surface of the steel plates was peened with a large number of impacts over a short period of time. The plastic deformation in the surface layer coupled with the large strain and high strain rate resulted into a progressive reduction of the original micro-scale grains into nanograins. The metastable austenite of the AISI 301 was partially transformed into martensite during this dynamic plastic deformation process. A 20-kHz ultrasonic transducer was exploited as the impulse source. The chamber diameter was 70 mm and the working distance between the steel plates and the horn surface was 15 mm. Both sides of the steel plates were SMAT treated to a total time of 30 min. More details of the set-ups, procedures and mechanism of the SMAT are described elsewhere[32-37].

For mechanical testing, treated samples were cut into dog-bone shapes with a gauge length of 30 mm and a width of 6 mm, and tested at room temperature at a strain rate of 6.7×10^{-4} s^{-1}. Seven specimens were tested to confirm repeatability. SEM and EBSD observations were performed on a JEM-6700F field-emission scanning electron microscope (SEM) equipped with an Oxford EBSD detector and HKL channel 5 software. TEM observations were carried out on a JEM-2100F transmission electron microscope with operating voltage of 200 kV. The plane-view TEM foils of the layers from certain depths were obtained by first polishing the corresponding surface layer, then mechanically polishing the sample from the untreated side until the sample reaches about the thickness of 30 μm. The treated side of the foil was protected with a resin and the foil was finally thinned down by electro-chemical polishing from the untreated side.

Theoretical Modeling

To provide fundamental basis to this experimental study, we developed a microstructure-based plasticity model to support our observations and to explain the mechanical performance of the hierarchical steel. When subjected to uniaxial tensile deformation, we can assume that the uniaxial uniform strain acts in every structural hierarchy of our steel sample. For the heterogeneous structures, the micromechanical approaches such as the self-consistent models[38-40] and revised mean-field methods[41-43] are usually applied to simulate the effective stress and strain by considering the interaction between the components. For the gradient structures, it has been proved that the rule of mixtures of Voigt model is a reasonable means to calculate the effective stress[44-46]. As such, the effective stress can be expressed as:

$$\tilde{\sigma}_{xx} = \frac{\sum_{i=1}^{n} {}_i\sigma_{xx}^{\mathrm{N}} H_i^{\mathrm{N}} + \sigma_{xx}^{\mathrm{C}} H^{C}}{H}, \tag{1}$$

where i, N, and C denote the ith layer, nanostructured region and coarse-grained region, respectively. n is the number of layers in the nanostructured region, ${}_i\sigma_{xx}^{\mathrm{N}}$ and σ_{xx}^{C} are the stresses applied on i^{th} layer of nanostructured and coarse-grained region, respectively, and $H_i^N = H/n$, H^{C}, H are the thickness of ith layer in nanostructured region, coarse-grained core, and the entire sandwich structured metal, respectively.

The stresses ${}_i\sigma_{xx}^{\mathrm{N}}$ and σ_{xx}^{C} in sandwich structured metal can be determined in the framework of the elasto-plasticity theory. The total strain rate $\dot{\boldsymbol{\varepsilon}}$ can be decomposed into its elastic and plastic parts: $\dot{\boldsymbol{\varepsilon}}^{(i)} = \dot{\boldsymbol{\varepsilon}}^{(i)\mathbf{e}} + \dot{\boldsymbol{\varepsilon}}^{(i)\mathbf{p}}$ .The elastic strain rate-stress rate follows the linear-elastic constitutive relation as $\dot{\boldsymbol{\varepsilon}}^{(i)\mathbf{e}} = \mathbf{M}^{(i)} : \dot{\boldsymbol{\sigma}}^{(i)}$, where $\mathbf{M}^{(i)}$ is the elastic compliance tensor of dual-phased grain. The plastic strain rate is proportional to the deviatoric stress $\boldsymbol{\sigma}^{(i)\prime}$, namely $\dot{\boldsymbol{\varepsilon}}^{(i)\mathrm{p}} = 3\dot{\varepsilon}^{(i)\mathrm{p}}\boldsymbol{\sigma}^{(i)\prime}/2\sigma_{\mathrm{e}}^{(i)}$. Here, ${\sigma^{(i)}_{ij}}' = \sigma^{(i)}_{ij} - \sigma^{(i)}_{kk}\delta_{ij}/3$, and $\sigma_e^{(i)} = \sqrt{3{\sigma^{(i)}_{ij}}'{\sigma^{(i)}_{ij}}'/2}$ is the von Mises equivalent stress. $\dot{\varepsilon}^{(i)\mathrm{p}}$ is the equivalent plastic strain rate which is determined by:

$$\dot{\varepsilon}^{(i)\mathrm{p}} = \dot{\varepsilon}^{(i)}\left[\frac{\sigma_{\mathrm{e}}^{(i)}}{\sigma_{flow}^{(i)}}\right]^{\mathrm{m}_0}, \tag{2}$$

where $\dot{\varepsilon}^{(i)} = \sqrt{2\dot{\varepsilon}^{(i)}_{ij}{}'\dot{\varepsilon}^{(i)}_{ij}{}'/3}$ is the equivalent strain rate and $\dot{\varepsilon}^{(i)}_{ij}{}' = \dot{\varepsilon}^{(i)}_{ij} - \dot{\varepsilon}^{(i)}_{kk}\delta_{ij}/3$. $\sigma_{flow}^{(i)}$ is the flow stress of the ith layer, and m_0 is the rate-sensitivity exponent. The nano-lamellae formed by the martensite are taken to be effective blocks to impede the movement of dislocations inside each grain, leading to dislocations pile-ups along the martensite/austenite interfaces. Consequently, the dislocation pile-up zones can be created near the interfaces between the nano-band martensite and nano-lamellar austenite. Thus, the flow stress in the grains containing nano-lamellae can be expressed as:

$$\bar{\sigma}_{flow}^{(i)} = \sigma_0 + M\alpha\mu b\sqrt{\rho_{GB} + \rho_{CI} + \rho_{Lame}^{(i)}}\,. \tag{3}$$

where σ_0 is the lattice friction stress, and $\boldsymbol{M}, \alpha$, μ , b are the Taylor factor, empirical constant, shear modulus and the Burgers vector, respectively, and ρ_{GB} is the density of dislocations in the dislocation pile-up zones along the grain boundaries (GBDPZ) when grain size achieves the nanometer scale, given as $\rho_{GB} = 6d_{GBDPZ}\eta^{GB}/bd_G$. Here, d_{GBDPZ} is the thickness of the GBDPZ, η^{GB} is the strain gradient in the GBDPZ, d_G is the grain size. ρ_{CI} is the density of dislocations in the interior of the crystal grains, determined by the Kock-Mecking model[42,47,48], and ρ_{Lame} is the dislocation density in the dislocation pile-up zones near the interfaces of the martensitic/austenitic nano-lamellae. By extending the derivation of the dislocation density in dislocation pile-up zones, that was successfully used in the description of the behavior of nano-twinned metals[49,50], to the situation for nano-lamellae boundaries in the present work, the dislocation density, ρ_{Lame}, can be expressed as:

$$\rho_{Lame}^{(i)} = \frac{1}{H_a + H_m}\left(\eta_0 H_a + \eta_1 - \eta_2 \frac{1}{H_a}\right) \;, \tag{4}$$

where $\eta_\alpha (\alpha = 0,1,2)$ are the constants. H_a is the thickness of austenite lamellae, and H_m is the thickness of hcp ε-martensite lamellae. For the coarse grains in the core, the overall flow stress can be expressed as:

$$\sigma_{CG}^{(i)} = \sigma_0 + M\alpha_0\mu b\sqrt{\rho_{CI}} + \sigma_B\,, \tag{5}$$

where σ_B represents the back stress. One can find from Eqs. (3) and (4) that the overall flow stress of the nano-lamellar structures can be directly associated with the density of dislocations in

dislocation pile-up zones, which is a function of the thickness of nano-lamellae and their depth from the top surface of the hierarchical steel.

Material parameters used in following calculations are extracted from the literature[46,51] or confirmed by fitting to the experimental results. Supplementary Table 1 lists the input parameters used in the model. In Supplementary Figure 1, the simulated constitutive relation of the hierarchical steel is calibrated against the experimental data, as are the gradient size scale of the microstructures and the dependence of yield strength on depth. It is apparent from Supplementary Figure 1 that the model successfully captures the stress-strain response of the hierarchical steel and the depth-dependent yield strength variations.

After carefully calibrating the model, we examined the differences of the nano-scale lamellar structure in the hierarchical steel with respect to the absence of any lamellar structure in classical gradient steels (described in the main text), as schematically illustrated in Fig. 2. Assuming that the gradient steel has a comparative microstructure with the same through-thickness grain-size variation but without nanoscale lamellae, we plot the predicted depth-dependent dislocation density in the hierarchical steel compared to that in the gradient steel in Supplementary Figure 2a. With exactly the same grain-size gradient, the depth-dependent dislocation density for the hierarchical steel with its nano-lamellae is ~4-10 times higher than that for the comparable classical gradient steel, as seen in Supplementary Figure 2a; correspondingly, the depth-dependent yield strength is up to 600 MPa higher within the layers containing nano-lamellae (down to a depth of ~300 μm below the surface) as shown in Supplementary Figure 2b. Supplementary Figure 2c illustrates the comparison of the overall stress-strain behavior between the hierarchical steel and the comparable classical gradient steel with the same grain size gradient, and clearly shows that due to the existence of the nano-lamellae, the yield strength and flow stress are both ~400 MPa or ~50% higher than that of the classical gradient steel.

To analyze the internal stress distribution during deformation, we further depict the predicted stress-strain curves in Supplementary Figure 3a for three separated regions in hierarchical steel from surface to core through-thickness (see inset of Supplementary Figure 3a and Supplementary Figure 2). It is apparent that the yield stresses in harder outer layers (Regions I and II) are much larger than that in the softer core layer (Region III); as such, the harder outer layers enhance the overall yield strength and flow stress of the hierarchical steel. For a sample

geometry with a 1 mm thickness and 100 mm width, we further plot in Supplementary Figure 3b the simulated total loading forces undertaken by each of the three regions as a function of the engineering strain. The harder outer layers (Regions I and II) can be seen to bear most of the loading forces as compared to the softer core layer (Region III). Due to the much higher average flow strength, the outermost layer (Region I) with the smallest nano-lamellae (0-100 μm depth), which occupies only ~20% of the overall volume, can sustain more than 50% of the overall load under uniaxial tension (Supplementary Figure 3a,b). Supplementary Figure 3c compares the average true stress-strain behavior of harder outer layers (Region I and II) in the hierarchical steel with that in the classical gradient steel. From this figure, it is apparent that because of the significantly higher load-bearing capacity of the outer layers (~300 μm in depth) in the hierarchical steel with its nano-scale lamellar microstructure, these outer layers can carry ~25% more of the applied load than the corresponding classical gradient microstructure at the same tensile plastic strain.

These micromechanics simulations speak to the following conclusions: 1) Assuming that the nano-scale lamellar interfaces serve as effective sources, sinks as well as strong barriers for dislocation motion during deformation, the graded nano-lamellar microstructure results in significant dislocation density increases within the hierarchical steel. More than a four-fold increase in dislocations densities within a ~300 μm depth (and about one order of magnitude increases within ~100 μm of the surface) are predicted for the hierarchical steel, as compared to that for a companion classical gradient steel without such a nano-lamellar structure. 2) Four to ten-fold higher dislocation densities and pile-ups along the nanoscale lamellar interfaces result in up to 600 MPa increase in yield strength and flow stress, and consequently a 25% increase in load-bearing capacity of the nano-lamellar structured surface layers (~300 μm in depth). Therefore, the addition of nanoscale lamellar structures can enhance the overall yield strength and flow stress of the hierarchical steel by 400 MPa when compared to the corresponding properties of a comparable gradient steel. 3) In addition, from insets of Supplementary Figure 1 and Supplementary Figure 2b, it is clear that the hierarchical steel has rather smooth depth-dependent changes in terms of yield strength, hardness and microstructure size-scale. These experimentally observed and theoretically validated smooth gradients help reduce stress or strain concentrations within the microstructure.

Additional References

31. J. Lu, Ordinary Metals - With a nano-scale boost, *Scientific American*, Paris ***VI,*** 21–22 (2015).
32. G. R. Huang et al., Analytical modelling for ultrasonic surface mechanical attrition treatment. *AIP Advances* 5, 077126 (2015).
33. W. P. Tong, N. R. Tao, Z. B. Wang, J. Lu, K. Lu, Nitriding iron at lower temperatures, *Science* ***299***, 686–8 (2003).
34. K. Y. Zhu, A. Vassel, F. Brisset, K. Lu, J. Lu, Nanostructure formation mechanism of α-titanium using SMAT, *Acta Mater.* ***52***, 4101–4110 (2004).
35. B. Arifvianto, M. Mahardika, P. Dewo, P. T. Iswanto, U. a. Salim, Effect of surface mechanical attrition treatment (SMAT) on microhardness, surface roughness and wettability of AISI 316L, *Mater. Chem. Phys.* ***125***, 418–426 (2011).
36. Y. Lin, J. Lu, L. Wang, T. Xu, Q. Xue, Surface nanocrystallization by surface mechanical attrition treatment and its effect on structure and properties of plasma nitrided AISI 321 stainless steel, *Acta Mater.* ***54***, 5599–5605 (2006).
37. H. Q. Sun, Y.-N. Shi, M.-X. Zhang, K. Lu, Plastic strain-induced grain refinement in the nanometer scale in a Mg alloy, *Acta Mater.* ***55***, 975–982 (2007).
38. H. Sabar, M. Berveiller, V. Favier, S. Berbenni, A new class of micro-macro models for elastic-visoplastic heterogeneous materials, *Int. J. Solids Struct.* ***39***, 3257-76(2002).
39. S.Berbenni, V. Favier, M. Berveiller , Impact of the grain size distribution on the yield stress of heterogeneous materials, *Int. J. Plasticity* ***23***, 114–42(2007).
40. X. Xiao, D. Song, J. Xue, H. Chu, H. Duan, A self-consistent plasticity theory for modeling the thermo-mechanical properties of irradiated fcc metallic polycrystals, *J. Mech. Phys. Solids* ***78***, 1-16 (2015).
41. G. J. Weng, A homogenization scheme for the plastic properties of nanocrystalline materials, *Rev. Adv. Mater. Sci.* ***19***, 41-62 (2009).
42. L. L. Zhu, S. Q. Shi, K. Lu, J. Lu, A statistical model for predicting the mechanical properties of nanostructured metals with bimodal grain size distribution, *Acta Mater.* ***60***, 5762-72 (2012).
43. L. L. Zhu, X. Guo, H. H. Ruan, J. Lu, Prediction of mechanical properties in bimodal nanotwinned metals with a composite structure, *Comp. Sci. Tech.* ***123***, 222–31 (2016).
44. J. Li, A.K. Soh, Modeling of the plastic deformation of nanostructured materials with grain size gradient, *Int. J. Plast.* ***39***, 88-102 (2012).
45. L. L. Zhu, C. S. Wen, C. Y. Gao, X. Guo, J. Lu, A study of dynamic plasticity in austenite stainless steels with a gradient distribution of nanoscale twins, *Scr. Mater.* ***133***, 49-53 (2017).
46. L. L. Zhu, H. H. Ruan, A. Y. Chen, X. Guo, J. Lu, Microstructures-based constitutive analysis of mechanical properties of gradient-nanostructured 304 stainless steels, *Acta Mater.* ***128***, 375-90 (2017).
47. L.L. Zhu, J. Lu, Modelling the plastic deformation of nanostructured metals with bimodal grain size distribution, *Int. J. Plasticity* ***30-31,*** 166-184 (2012)..
48. U.F. Kocks and H. Mecking, Physics and phenomenology of strain hardening: the FCC case, *Prog. Mater. Sci.* ***48***, 171-273 (2003).
49. L.L. Zhu, H.H. Ruan, X.Y. Li, M. Dao, H.J. Gao, J. Lu, Modeling grain size dependent optimal twin spacing for achieving ultimate high strength and related high ductility in nanotwinned metals, *Acta Mater.* ***59,*** 5544-5557 (2011).
50. L.L. Zhu, S.X. Qu, X. Guo, J. Lu, J. Analysis of the twin spacing and grain size effects on mechanical properties in hierarchically nanotwinned face-centered cubic metals based on a mechanism-based plasticity model, *J. Mech. Phys. Solid.* ***76***,162-179 (2015).

51. S. L. Wong, M. Madivala, U. Prahl, F. Roters, D. Raabe, A crystal plasticity model for twinning-and transformation-induced plasticity, *Acta Mater*. ***118***, 140-151(2016).

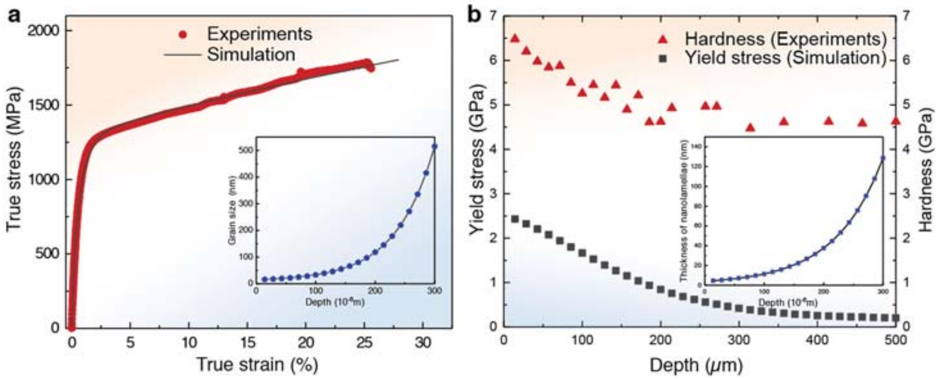

Supplementary Figure 1 | Calibration of the numerical model in terms of the experimental stress-strain curve and hardness gradient of the hierarchical steel. a. The simulated true stress-strain curve is compared with the experimental data; inset shows the grain size variation within the nano-lamellae structure (down to a ~300 μm depth), consistent with experimental observations. **b.** The experimental hardness data and the calibrated yield stress as a function of the depth below the surface for the hierarchical steel; inset shows the depth dependent nano-lamellae thickness variation within the nano-lamellae structure (down to a ~300 μm depth), again consistent with experimental observations.

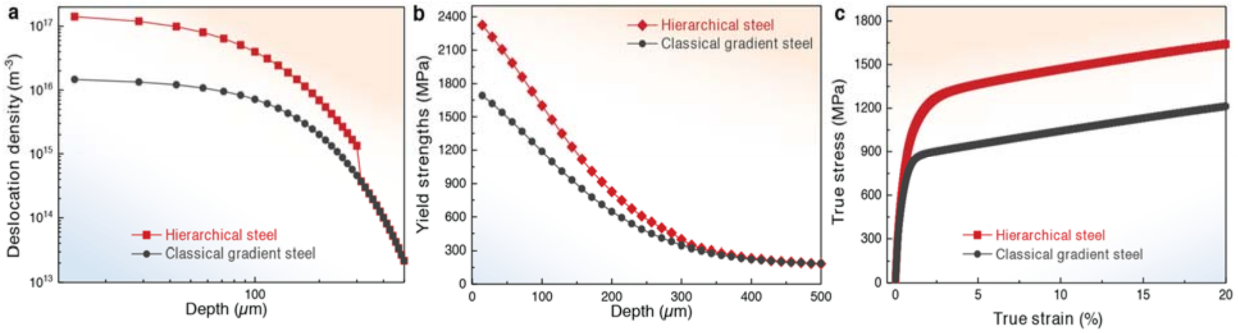

Supplementary Figure 2 | Comparison of dislocation densities and mechanical properties for the hierarchical steel and the comparable classical gradient steel, predicted by the experimentally calibrated micromechanics model. a. The predicted depth-dependent dislocation densities. **b**. The predicted through-thickness yield strength variations. **c**. The predicted stress-strain behavior.

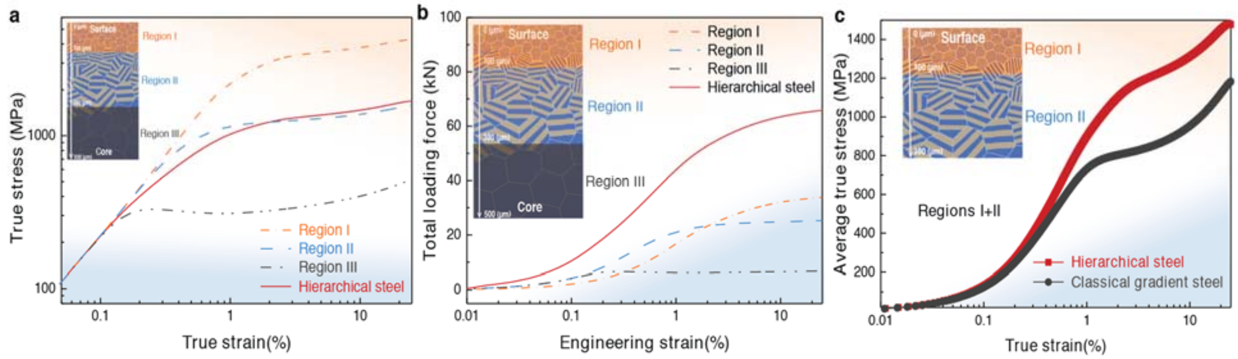

Supplementary Figure 3 | Load-bearing capacity for the nano-lamellar structured layers and the core layer. a. The true stress-strain curves of the overall hierarchical steel and of the three separate regions from surface to core. Inset in **a** schematically illustrates the three separate regions named Region I (~100 μm in depth), Region II (~100-300 μm in depth) and Region III (~300-500 μm in depth), respectively, in the hierarchical steel from surface layer to core area. **b**. The predicted total loading force-engineering strain curves for the overall hierarchical steel as well as for each of the three separate regions within the hierarchical steel. **c**. The average true stress-strain behavior for Region I + Region II (~300 μm in depth) in the hierarchical steel is compared with that in the comparable classical gradient steel without nano-lamellae.

Supplementary Table 1.

Description, symbol, magnitude and equation in which the different parameters of the models appear.

Parameter (Unit)	Symbol	Magnitude
Elastic constants (GPa) (austenite)	C11,C12,C44	175;115;135
Elastic constants (GPa) (hcp martensite)	C11,C12,C13; C33;C44	242 ; 118;45 ;315 ;41
Poisson's ratio	ν	0.29
Magnitude of the Burgers vector (nm)	b	0.26
Taylor factor	M	3.06
Taylor constant	α	0.3
Thickness of GBDPZ (nm)	d_{GBDPZ}	2.898
Strain gradient in GBDPZ (m^{-1})	η_{GB}	3.5x10^6
Dislocation density parameter (m^{-3})	η_0	1.5x10^6
Dislocation density parameter (m^{-2})	η_1	0.075
Dislocation density parameter (nm^{-1})	η_2	0.15
